# The clinical relevance of hyper-reflective foci in the inner retina at the diagnosis of multiple sclerosis

**DOI:** 10.1186/s42466-025-00447-3

**Published:** 2025-11-14

**Authors:** Marco Puthenparampil, Alessandro Miscioscia, Elisa Basili, Valentina Annamaria Mauceri, Marta Pengo, Tommaso Torresin, Federica De Napoli, Elisabetta Pilotto, Francesca Rinaldi, Paola Perini, Edoardo Midena, Paolo Gallo

**Affiliations:** 1 Multiple Sclerosis Centre, Department of Neuroscience, Università degli Studi di Padova, Padova, Italy; 2 Day Hospital and Advanced Therapy in Neurological Disorders, Neurology Clinic, Azienda Ospedaliera di Padova, Padova, Italy; 3 Ophthalmology Clinic, Azienda Ospedaliera di Padova, Padova, Italy

**Keywords:** Multiple Sclerosis, Optical Coherence Tomography, Hyperreflective Foci, Disease activity, Prognostic factor

## Abstract

**Background:**

Hyper-Reflective foci (HRF) increased in the inner retina (IR) of patients with Multiple Sclerosis (pwMS).

**Objective:**

To evaluate the risk of therapeutic failure, based on HRF count at baseline.

**Methods:**

Fifty-seven pwMS were included in this retrospective, cohort, single-centre study. All patients were enrolled at clinical onset and were treatment-naive, with no evidence of optic nerve involvement. Patient were divided at baseline based on the MS treatment in platform-therapy pwMS (PTpwMS) and as High efficacy therapy pwMS (HETpwMS). Then, all PTpwMS were followed up (87.6±31.2 months) to evaluate the time to therapeutic switch for lack of efficacy on outcomes. HRF count was expressed as the sum of both eyes in Ganglion Cell-Inner Plexiform Layer (GCIP), Inner Nuclear Layer (INL) and Inner Retina (IR, GCIP + INL).

**Results:**

Survival analysis confirmed an increased risk of therapeutic switch in those patients with a higher HRF-INL count (Log-Rank *p* < 0.0001, H.R. 7.9, _95%_CI 2.6–24.5). PTpwMS switching during the follow up had significantly higher HRF count in INL compared to not-switching (45.80 ± 10.32vs 31.75 ± 6.27, *p* < 0.05).

**Conclusions:**

HRF might be a useful marker to predict the risk of acute demyelination in MS and might give help Neurologist in therapeutic decision.

**Supplementary Information:**

The online version contains supplementary material available at 10.1186/s42466-025-00447-3.

## Introduction

Multiple Sclerosis (MS) is a chronic inflammatory and neurodegenerative disorder of the Central Nervous System (CNS). In white matter pathology, a pivotal detrimental mechanism consists in the acute inflammation inside the CNS, that is driven by the acute recruitment of adaptive-immunity cells. These cells enter the CNS and target CNS-specific antigens on myelin [1] causing demyelination, the hallmark of MS [2]. Microglia cells (MGs), CNS resident macrophages, might influence the activity of adaptive immunity in many steps: first, MGs might act on blood–brain barrier (BBB), enhancing immune-cell diapedesis [3]; second, it is a professional antigen-presenting cell, thus able to present CNS cels-antigen on MHC-II molecules, but also on MHC-I molecules (cross-presentation), enhancing T-cell activity [4]; third, MGs can secrete cytokines that support B and T-cell activity [5]; finally, a role for MGs has been recognized also in the persistent chronic inflammation that characterize MS lesions [6]. The rate of lesion accumulation is particularly relevant in early disease phase, when disability correlates with relapse associated worsening [7]. Therefore, the identification of MGs activity might help estimate the level of adaptive-immunity recruitment in the CNS [8]. Optical Coherence Tomography (OCT) macular scans are able to identify small hyperreflective foci (HRF, called hyper-reflective spots or dots) [9, 10], which might be the expression of activated MGs, as indicated by animal models [10], as well as by the analysis of aqueous humour in intractable macular oedema and of cerebrospinal fluid in MS [11, 12]. In MS, a significant increase of HRF count was observed, particularly associated with inflammatory disease activity [8, 13]. Since their prognostic role is still debated, we evaluated in a cohort of pwMS at clinical onset the HRF count whether the baseline HRF count predicted the risk of disease activity.

## Materials and methods

### Study population: inclusion and exclusion criteria

Relapsing-Remitting MS (pwMS) at clinical onset between January 2014 and June 2020 were enrolled in this single centre, longitudinal, retrospective cohort-study. Inclusion criteria were: (1) diagnosis of RRMS achieved according to the revised 2017 McDonald criteria [14]; (2) disease duration (defined in this study as the interval between the first clinical symptom attributable to MS and the date of the OCT evaluation) < 18 months; (3) age between 18 and 60 years; (4) the administration of an MS-specific treatment in line with the Italian Medicines Agency (Agenzia Italiana del FArmaco, AIFA) guidelines. Exclusion criteria were: (1) systemic and ophthalmologic disorders (such as diabetes); (2) diagnosis of progressive MS; (3) steroid therapy in the month prior to OCT acquisition; (4) previous clinical history of optic neuritis; (5) evidence of clinical or subclinical optic neuritis (inter-eye difference in peripapillary Retinal Nerve Fibre Layer (pRNFL) of >5% and/or macular Ganglion Cell-Inner Plexiform (mGCIP) Layers inter-eye difference of 4%, and/or optic nerve hyperintensity in ≥ 2 slices at Brain MRI Double Inversion Recovery Sequence) [15]. The study was conducted in agreement with the Declaration of Helsinki and approved by the local Ethic Committee (Comitato Etico per la Sperimentazione Clinica dell’Azienda Ospedaliera di Padova, prot.n. 17760).

### OCT and HRF count

**Fig. 1:**
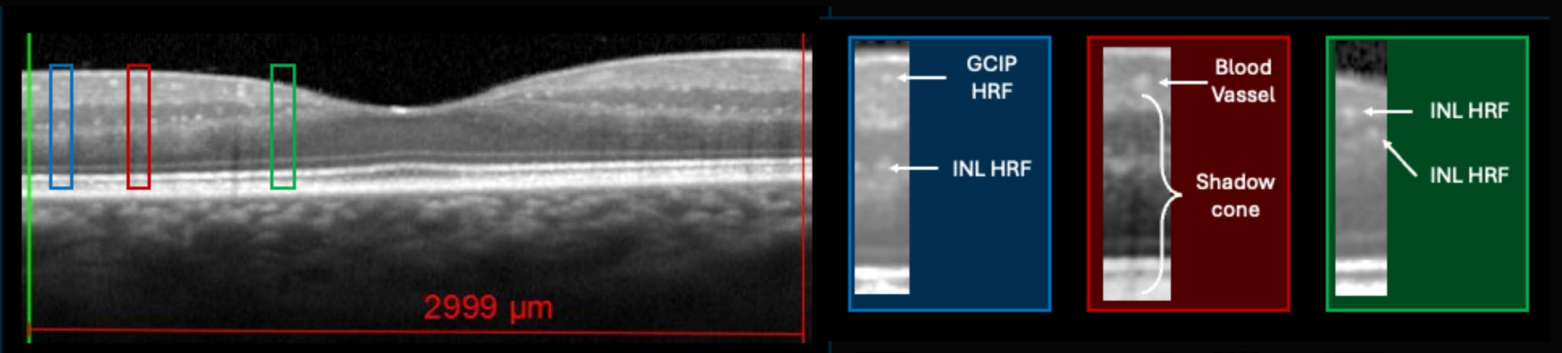
HRF count in GCIP, INL and IR. HRF were counted and divided based on retinal in GCIP and INL. IR HRF count consists in the sum of both layers. Boxes show magnified images, whose contrast and brightness had to be modified. Created in BioRender. Puthenparampil, M. (2025) https://BioRender.com/2bd2brp

All patients performed at baseline OCT (for HRF count, as already explained) in line with OSCAR-IB and APOSTEL criteria [16, 17]. MS patients underwent spectral-domain OCT (Spectralis; Heidelberg Engineering, Carlsbald, CA; Heidelberg Eye Explorer version 1.7.0.0) examination of both eyes in a dark room without the injection of any mydriatic agent. The peripapillary 3.4 mm ring scan, centered on the optic nerve head, was used to measure mean global pRNFL and mean sectorial peripapillary thickness (temporal pRNFL, pRNFL-T; supero-temporal RNFL, pRNFL-TS; supero-nasal RNFL, pRNFL-NS; nasal RNFL, pRNFL-N; infero-nasal RNFL, pRNFL-NI; infero-temporal RNFL, pRNFL-TI). Moreover, mean thickness of the papillomacular bundle (pRNFL-PMB) was also measured. Scans with ART between 95 and 100 frames were considered valid. The macular scan, automatically centered on the fovea, was composed of vertical 25 linear scans in the High-Resolution Mode and 49 ART. The retinal layering was obtained using the automatic layering of the Spectralis SD-OCT as previously published [18]. The following retinal slabs, automatically obtained by the incorporated algorithm, were measured in the vertical macular map: macular RNFL (mRNFL); macular ganglion cell layer (mGCL); macular inner plexiform layer (mIPL); macular inner nuclear layer (mINL); macular outer plexiform layer (mOPL); macular outer nuclear layer (mONL). The mGCL and mIPL were then unified in a unique layer (mGCIP). The analysis of the central linear scan of the macular map (ART 100), crossing the fovea (single horizontal scan), was considered for HRF counting. HRF were counted in the area included between two perpendicular lines to Brunch’s membrane traced at 1500 μm both temporally and nasally from the centre of the fovea only when scan quality index was >30. HRF were defined as isolated, small size (< 30 μm), punctiform elements with moderate reflectivity (similar to that of the nerve fibre layer) but without any back shadowing. The count was performed in GCIPL and INL separately; results were reported also as Inner Retina (IR) HRF count (i.e., GCIP HRF count + INL HRF count)(Fig. 1). The presence of HRF was rated by two independent blind observers (MaPe, TT) by consensus [8, 19]. The count was performed in the GCL, IPL and INL separately and results were reported also as GCIP HRF count (i.e., GCL HRF count + IPL HRF count) and as inner retina (IR) count (i.e., GCIP + INL count). HRF in GCIP, INL, and inner retina (IR) were counted and reported for analysis as the sum of both eyes. The identification of asymptomatic optic nerve involvement required also the evaluation of inter-eye difference of pRNFL and mGCIP [15].

### Clinical and radiological follow-up

All patients were clinically and radiologically followed up [20]. Trained neurologists (MP, FR, PP, and PG) indicated the EDSS score, at baseline and then every 6 months evaluated the presence of a clinical/radiological relapse and indicated the therapeutic switch. A clinical relapse was defined as the occurrence of new symptoms or exacerbation of existing symptoms that lasted for 24 h or longer, in the absence of concurrent illness or fever, and occurring 30 days or more since a previous relapse [21]. MRI activity was defined in presence of contrast-enhancing lesions on T1-weighted images or new/enlarging hyperintense lesions on T2-weighted images (compared to the baseline scan), after checking the fluid-attenuated inversion recovery sequence. MRI was performed at least every 12 months in all pwMS.

### Statistical analysis

Group differences between patients were tested by Chi-square test for sex, by T-test for age, by U-test for EDSS. For assessing the relationship between therapeutic switch (due to inflammatory reactivation) during the follow up and OCT parameters, linear mixed-effects models were used with the retinal measure as the dependent variable, the reactivated/non-reactivated group as independent variable, and participants as random effect to account for within-patient inter-eye correlations as recommended [16]. The cut-off for disease activity prediction was calculated with ROC curve comparing the HRF count in patients starting platform-therapy (PTpwMS) switching vs. not switching. The cut-off was then confirmed in a survival analysis (log-rank test). For multiple comparison analysis (HETpwMS vs. switching PTpwMS, and not switching PTpwMS), Kruskal-Wallis test with Dunn’s correction was performed. IBM SPSS (v29) was used for all the analysis. A *p*-value lower than 0.05 was considered statistically significant.

## Results

### Study population

Fifty-seven pwMS were enrolled in this study. All patients performed MRI annually and a complete neurological evaluation every 6 months. At baseline, 9 patients had the clinical criteria to start a HET (HETpwMS), while 48 patients to start a platform therapy (PTpwMS). Among them, 12 patients (25%) experienced at least one clinical relapse, 22 (45.8%) had at least one radiological reactivation, and in 15 PTpwMS the therapy switch was then indicated. In presence of clinical relapse MRI always showed disease activity. The cohort of PTpwMS was followed up for 87.6±31.2 months (range 11–126). Among baseline clinical values, multiple comparison analysis did not reveal any difference in terms of EDSS and age at onset (Table 1). In PTpwMS pRNFL was evaluated in 95/96 eyes (99%), macular volumes in 89/96 eyes (93.8%), and HRF in 90/96 eyes (92.7%). In all HETpwMS macular volume were evaluated (18/18 eyes), while HRF were counted in 17/18 eyes (94.4%) and pRNFL was evaluated in 15/18 eyes (83.3%).

**Table 1:** Clinical and demographic variables at baseline

	PTpwMS switching (15 patients)	PTpwMS NOT switching (33 patients)	HETpwMS (9 patients)	*p*-value
***Gender ratio (F/M)***	1.5 (9/6)	2.3 (23/10)	0.4 (4/5)	0.376^a^
***EDSS***	2.0 (1.0–3.0)	2.0 (1.0-3.5)	2.0 (1.5-3.0)	0.024^b^
***Disease Duration (m)***	4 (0–18)	7 (0–10)	3 (0–12)	0.697^b^
***Time from Last relapse*** ^***c***^ ***(m)***	4 (0–17)	7 (1–10)	3 (1–12)	0.413^b^
***Age at onset (y)***	39.2 ± 11.8	31.0 ± 10.8	29.8 ± 5.6	0.014^b^
***PLATFORM THERAPY***				
*Interferon-β1*	3	8	0	0.5758 ^a^
*Glatiramoid*	2	10	0	
*Teriflunomide*	2	5	0	
*DimethilyFumarate*	7	10	0	
***HET THERAPY***				
*Natalizumab*	0	0	8	n.a.
*Alemtuzumab*	0	0	1	

Disease duration and time from last relapse are expressed in months (m), while age in years (y). Multiple comparison did not reveal any difference in terms of EDSS and age between groups. ^a^: Chi square test; ^b^: Kruskal Wallis test with dunn’s correction; c: time between last clinical or radiological relapse and OCT; abbreviations: HET: High-Efficacy Therapy; EDSS: expanded disability status scale

### In PTpwMS therapeutic switch associated with INL-HRF count at baseline

Regression analysis showed that therapeutic switch during the follow-up associated only with HRF count in INL and IR (Table 2).

**Fig. 2:**
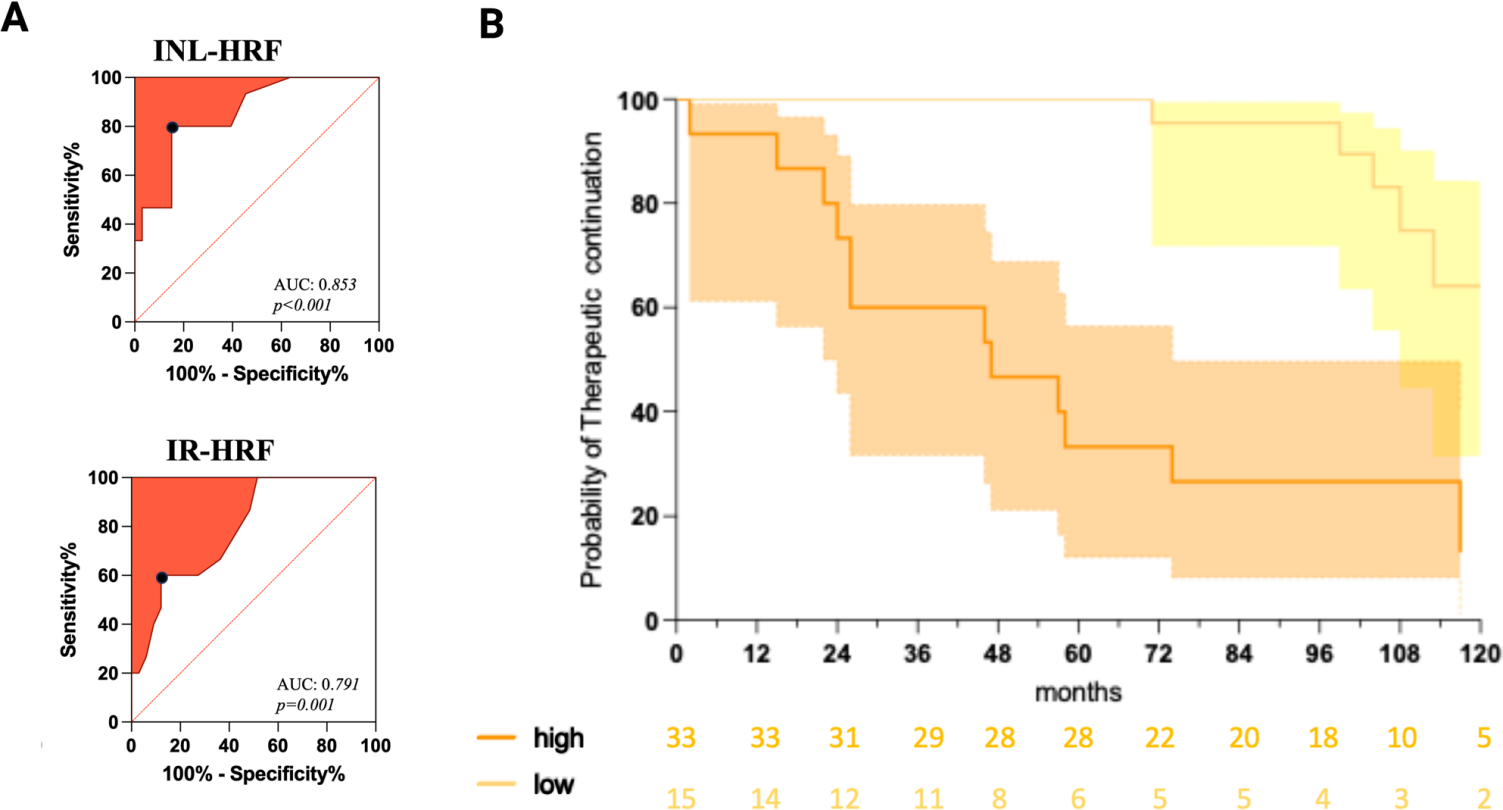
Therapeutic switch was predicted by HRF INL count at baseline. (**A**) ROC analysis identified the best cunt cut-off in INL (39 HRF), and IR (65 HRF) in PTpwMS. (**B**) Survival Analysis on PTpwMS classified based on HRF-INL count into high (orange) and low (yellow). Created in BioRender. Puthenparampil, M. (2025) https://BioRender.com/pt4mb9x

ROC analysis disclosed a best cut-off of 39 for HRF-INL count (sensitivity 80%, specificity 84.9%, Likelihood ratio 5.20, AUC 0.85, *p* < 0.001) (Fig. 2A). Survival analysis confirmed an increased risk of therapeutic switch in those patients with a higher HRF-INL count (Log-Rank *p* < 0.0001, H.R. 7.9, _95%_CI 2.6–24.5)(Fig. 2B).

**Table 2:** OCT parameters and inflammatory disease reactivation during the follow-up. a: macular volumes, expressed in mm3; b:RFNL thickness, expressed in µm; c: HRF count expressed as the mean of two eyes

	b-value	p-value
mRNFL^a^	-0.55	0.230
mGCL^a^	-0.24	0.642
mIPL^a^	-0.24	0.740
mGCIP^a^	-0.13	0.674
mINL^a^	0.12	0.885
mOPL^a^	-1.37	0.051
mONL^a^	-0.48	0.071
mRPE^a^	-0.33	0.811
pRNFL-G^b^	0.01	0.813
pRNFL-PMB^b^	0.01	0.870
pRNFL-T^b^	-0.01	0.717
pRNFL-TS^b^	0.01	0.532
pRNFL-TI^b^	-0.01	0.216
pRNFL-N^b^	0.01	0.582
pRNFL-NS^b^	0.01	0.818
pRNFL-NI^b^	0.01	0.267
HRS-GCIP^c^	0.02	0.267
HRS-INL^c^	0.05	0.001
HRS-IR^c^	0.03	0.001

Cox regression model confirmed the association between therapeutic switch risk and HRF-INL cut off (β:27.40, _95%_CI 6.16–210.0, *p* < 0.0001). Since therapeutic switch was indicated by clinical relapse and/or radiological disease activity, the association between demographic, clinical and HRF count was also evaluated by means of Cox regression analysis. While the relapse risk associated with the age at onset (β 0.93, _95%_CI 0.87–0.98, *p* = 0.012), radiological relapse risk associated with HRF-INL count (β 3.03, _95%_CI 1.30–7.21, *p* < 0.001) (Table 3).

**Table 3:** Baseline risk factor of therapeutic switch, clinical relapse, and radiological activation. Cox regression analysis was performed on PTpwMS. Abbreviations as in Table 1 and 2

	Univariate Analysis			Multivariate Analysis		
C. Therapeutic switch	H.R.	_95%_CI	p-value	H.R.	_95%_CI	p-value
Age at onset	0.955	0.905 – 1.001	0.068			
EDSS at baseline	1.828	0.726 – 4.200	0.176			
Disease duration at baseline (m)	1.000	0.877 - 1.117	0.996			
Gender	0.731	0.261 – 2.195	0.555			
HRF-INL	1.088	1.043 – 1.136	<0.0001	27.40	6.164 - 210.0	<0.0001
HRF-IR	1.071	1.032 - 1.112	0.0003			
A. Clinical Relapse	H.R.	_95%_CI	p-value	H.R.	_95%_CI	p-value
Age at onset	0.9257	0.867 - 0.979	0.012			
EDSS at baseline	1.312	0.462 - 3.317	0.590			
Disease duration at baseline (m)	0.968	0.833 - 1.094	0.632			
Gender	1.106	0.348 - 4.149	0.869			
HRF-INL	1.843	0.576 - 5.892	0.290			
HRF-IR	1.356	0.362 - 4.306	0.619			
B. Radiological Relapse	H.R.	_95%_CI	p-value	H.R.	_95%_CI	p-value
Age at onset	0.962	0.922 – 1.000	0.058			
EDSS at baseline	1.736	0.864 - 3.299	0.106			
Disease duration at baseline (m)	0.964	0.862 – 1.059	0.480			
Gender	1.068	0.449 - 2.805	0.886			
HRF-INL	3.026	1.299 - 7.207	0.010	3.026	1.299 - 7.207	0.010
HRF-IR	2.475	1.017 – 5.761	0.038			

### HRF count at baseline did not differ between PTpwMS switching and HETpwMS

**Fig. 3:**
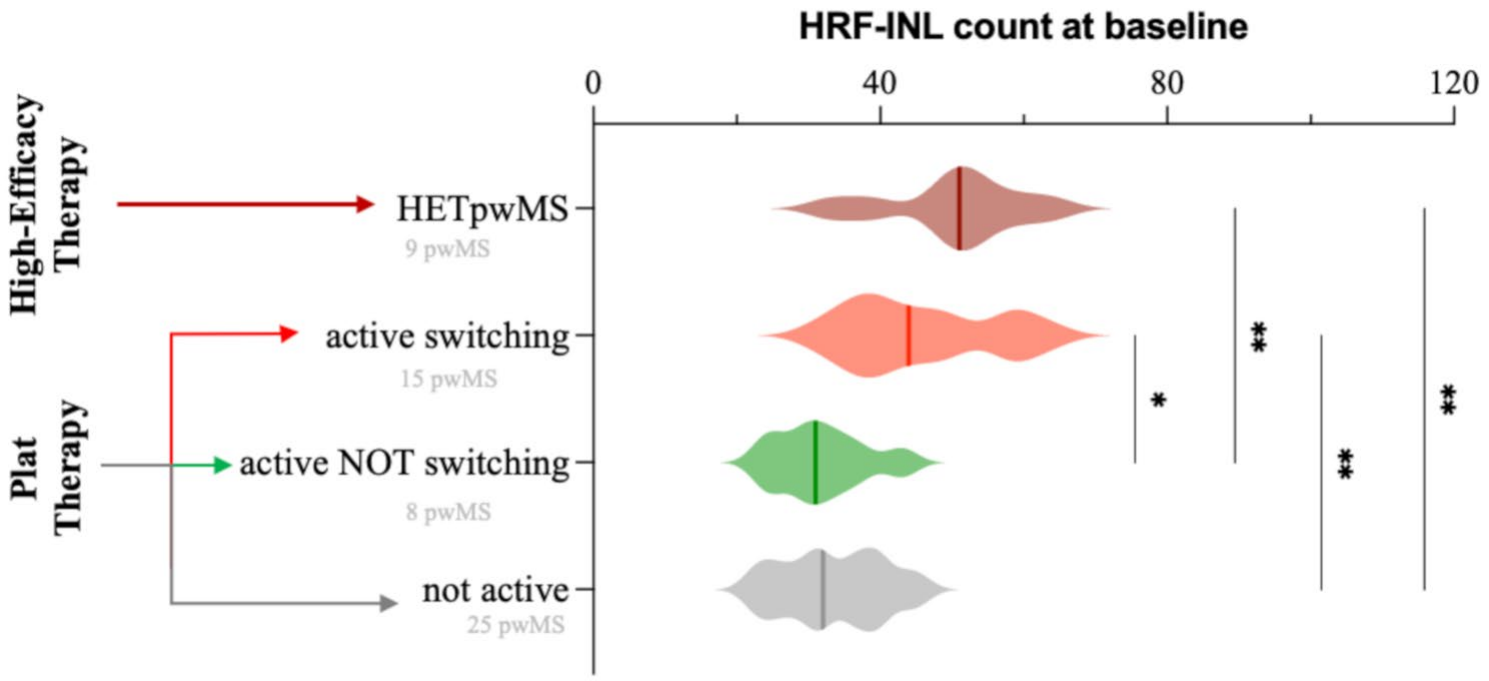
HRF count in pwMS subgroups. Compared to NOT switching or not active PTpwMS, HRF count was significantly higher in the INL, in both switching PTpwMS and HETpwMS. Created in BioRender. Puthenparampil, M. (2025) https://BioRender.com/9khhy6t

Based on disease activity (clinically and/or radiologically) and the therapeutic switch observed during the follow up, PTpwMS were divided into active switching (15 PTpwMS), active not switching (8 PTpwMS) and not active (thus not switching, 25 PTpwMS). OCT Data are reported in Supplementary Table. At baseline INL-HRF count was increased in HETpwMS (50.22 ± 9.44) compared with both not active PTpwMS (33.56 ± 7.01, *p* < 0.005) and active and not switching PTpwMS (31.75 ± 6.27, *p* < 0.005) Also between active and switching PTpwMS (45.80 ± 10.32) and both not active (*p* < 0.005) active and not switching (*p* < 0.05)PTpwMS (Fig. 3) (Supplementary Figure). ROC analysis disclosed that HRF INL count were able to predict not switcher PTpwMS patients (AUC 0.845, *p* < 0.001), identifying a cut-off of 45 (sensitivity 56.5%, _95%_IC 31.6% − 78.6%; specificity 94.1%, _95%_IC 75.0% − 99.6%).

## Discussion

The identification of strong, feasible, prognostic markers of inflammatory clinical and radiological disease progression is a pivotal in Multiple Sclerosis research [22–25]. Although activated MGs might play pivotal role in chronic disease phase and risk of disease progression, recent evidences further link MGs to acute CNS inflammation [26]. OCT allows the visualization in vivo of HRF, an in-vivo biomarkers of activated MGs [10, 12]. In line with these findings, we have already described a strong association between the presence of gadolinium-enhancing lesions and HRF-count, as well as an increased risk of radiological relapse and No Evidence of Disease Activity condition [8, 13]. In this project, we focused on the prognostic role of HRF count, working in a typical clinical practice, just analyzing the linear scan for HRF count and summing the foci of both eyes. Since the dynamic of HRF after optic neuritis is still unclear [27, 28], we decided to focus on patients with no evidence of optic nerve involvement, retrospectively evaluating the presence of optic nerve lesion, doth directly, excluding those patients with positive MRI (DIR sequences), and indirectly, with significant inter-eye asymmetry (pRNFL >5%, mGCIPL >4%), in line with most recent indications [29].

To weight the clinical relevance of OCT parameters, we evaluated the therapeutic choice of a group of pwMS followed at our MS centre and retrospectively collected the clinical evaluation and MRI reports of those patients starting platform therapy (PTpwMS), to distinguish those patients with evidence of inflammatory disease activity during the follow up. Interestingly and in line with previous findings, HRF INL count was associated to the risk of inflammatory disease reactivation [8]. Cox regression analysis revealed that age at onset predicted the risk of clinical relapse activity, a finding in line with the literature [7, 30], supporting the concept that younger patients are more probably in an inflammatory disease phase than elder patients. The low number of clinically relapsing patients requires a larger validation cohort to evaluate the relevance of other variables.

Even in our study the clinical relapse was the “tip of the iceberg”, since only 12 patients experienced a clinical relapse (25.0% of our patients) and 22 (45.8%) a radiological relapse. In these latter patients, an association with HRF-INL count was identified, driving the association also with the risk of therapeutic switch. These findings are in line with our previous report [8], but are obtained with a more basic method, expressing HRF count as the sum of HRF in both eye in a specific retinal layer, an activity that can be done during the clinical evaluation or, in a near future, indicated in the OCT report automatically [31].

Although the basic method to count the prognostic relevant INL-HRF count gives strength to our study, limitations are still present. First, the absence of a second validation cohort. However, the relevance of a prognostic biomarkers should be validated in multicenter, rather than monocentric, study. The observational, longitudinal prospective multicenter study would overcome also the additional limitations of our research, i.e. sample size and retrospective study-design. The possible validation of automatic algorithm to count HRF in the inner retina might facilitate the design and organization of these studies. Even if it is not related to our project, an additional limitation might hamper the diffusion of HRF count in clinical practice consists in the sequence required to count HRF. Indeed, in MS protocols since OCTMIS trial the macula has been studied with vertical linear scans (28 or 48 lines, 48 ART) [32], while HRF count requires a horizontal macular scan of at least 48ART (> 90ART and > 30 quality index are recommended, especially for automatic segmentation algorithms). Finally, follow-up data are not available. However, since different drug might act on HRF differently [18], the evaluation of HRF in pwMS under disease-modifying treatments should be evaluated separately.

In our cohort we have not confirmed the association between INL and new white matter lesion volume [33]. However, our cohort had less patients and the radiological activity we observed did not determine the accumulation of a high number of new/enlarging lesions, determining a narrowed range of new white matter lesion count. Moreover, since we specifically focused on patients with no evidence of optic nerve involvement, to avoid possible effect on HRF count, also to the absence of both symptomatic and asymptomatic optic nerve involvement might influence into the variability of both peripapillary and macular OCT measures [34–36]. This consideration could at least partially explain the narrowed variability in OCT and MRI parameters we observed: larger cohort with less restrictive inclusion criteria might help to better weight further associations. Nevertheless, a mild INL increase was observe also in our cohort in those pwMS with high inflammatory load, thus starting HET at onset. This data is in line with our previous observation that INL volume associates HRF INL in pwMS, further suggesting that an increased BRB permeability induced by microglial production of pro-inflammatory cytokines (IL-1 and IL-6) and iNOS might induce INL volume increases due to an impaired capacity of the Muller cells to maintain retinal fluid homoeostasis [37]. Indeed, an association with microcystic macular edema (MME) and HRF was already observed [38]. However, none of our patients had MME, since our patients were young, with short disease history and with no evidence of optic nerve involvement, which are all factors strongly associated (especially optic nerve history) with MME [39].

In conclusions, our study provides evidence (Level C) that HRF count at baseline might have a prognostic role in identifying these pwMS requiring a HET since early disease phase, helping neurologists in the therapeutic choice. Moreover, HRF in the inner retina can be linked again to acute CNS inflammation, supporting the tight interaction between resident microglia and infiltrating adaptive immunity. However, the temporal dynamic of microglia activation and adaptive immunity recruitment (i.e. outside-inside vs. inside-outside model) is still unclear [40]. Moreover, whether OCT might see chronic disease mechanisms in vivo and at disease onset is worth of interest and merits to be explored.

## Supplementary Information

Below is the link to the electronic supplementary material.

Supplementary Figure: Baseline HRF count at baseline within PwMS subgroups. HRF count in INL and IR was higher in active switching and HET pwMS than in not active and active no switching PTpwMS

Supplementary Table: Macular volumes and pRNFL thickness at baseline. ^a^: macular volumes, expressed in mm^3^; ^b^:RFNL thickness, expressed in µm. GEE did not identify any significant difference between groups

## Data Availability

Data are available upon reasonable request.
