# Supplementary figures and images for: The clinical relevance of hyper-reflective foci in the inner retina at the diagnosis of multiple sclerosis
Source: Neurol Res Pract. 2025 Nov 14;7(1):90. doi: 10.1186/s42466-025-00447-3 (PMC12619398; doi:10.1186/s42466-025-00447-3)

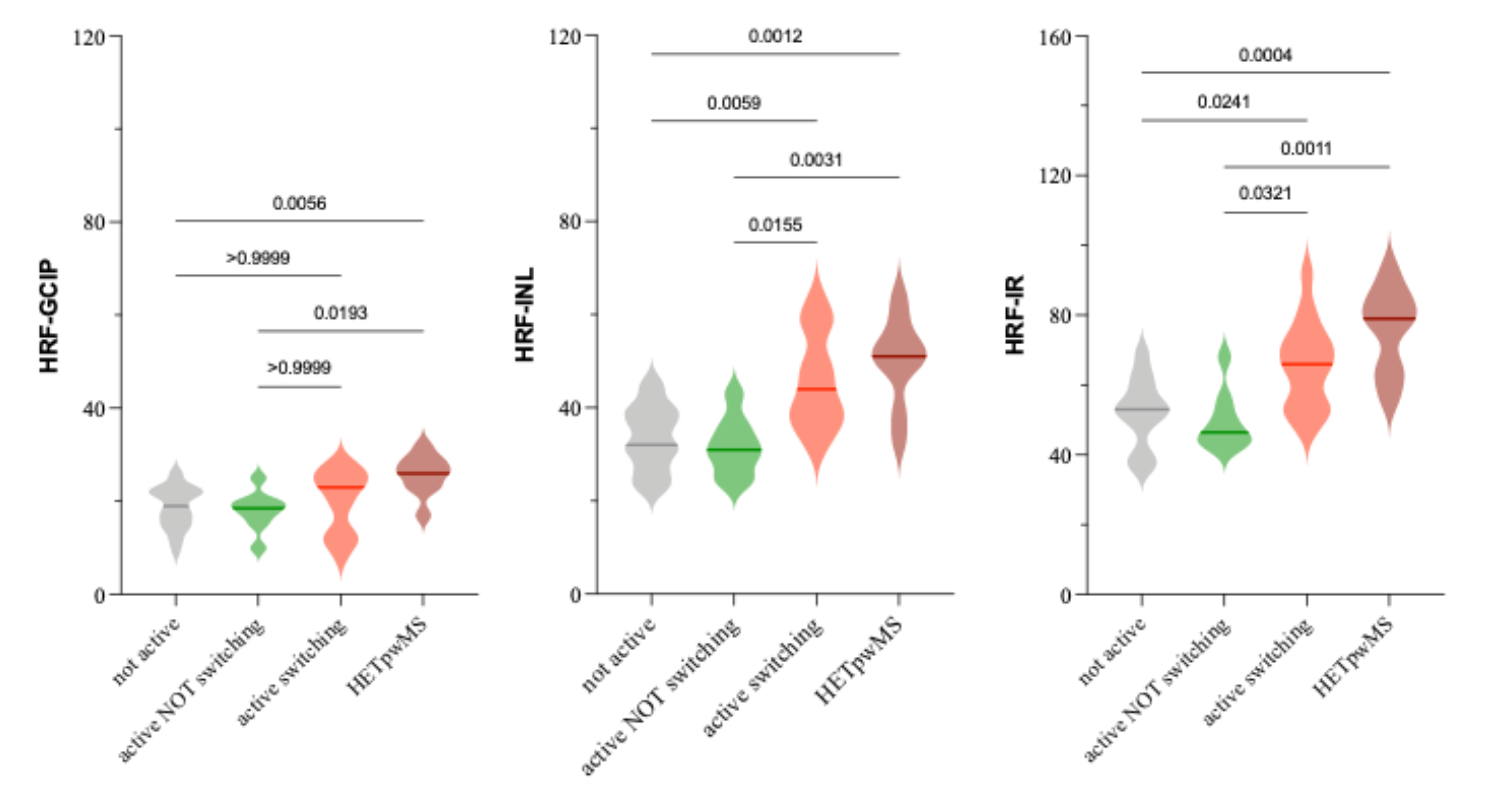

Supplement: Supplementary file 1 — Supplementary Figure: Baseline HRF count at baseline within PwMS subgroups. HRF count in INL and IR was higher in active switching and HET pwMS than in not active and active no switching PTpwMS [file 42466_2025_447_MOESM1_ESM.jpeg]
