# Supplementary material for: The clinical relevance of hyper-reflective foci in the inner retina at the diagnosis of multiple sclerosis
Source: Neurol Res Pract. 2025 Nov 14;7(1):90. doi: 10.1186/s42466-025-00447-3 (PMC12619398; doi:10.1186/s42466-025-00447-3)
Supplement: Supplementary file 2 — Supplementary Table: Macular volumes and pRNFL thickness at baseline. a: macular volumes, expressed in mm3; b:RFNL thickness, expressed in µm. GEE did not identify any significant difference between groups [file 42466_2025_447_MOESM2_ESM.docx]

**Supplementary Table. Macular Volumes and pRNFL Thickness at baseline.** ^a^: macular volumes, expressed in mm^3^; ^b^:RFNL thickness, expressed in µm. GEE did not identify any significant difference between groups.

|  | **not switching PTpwMS** | **Active not switching PTpwMS** | **Active switching PTpwMS** | **HETpwMS** |
| --- | --- | --- | --- | --- |
| **RNFL_TV^a^** | 0.9042 ± 0.1113 | 0.9250 ± 0.1518 | 0.8879 ± 0.0735 | 0.8839 ± 0.08813 |
| **GCIPL_TV^a^** | 2.071 ± 0.1541 | 2.100 ± 0.1614 | 2.061 ± 0.1671 | 2.051 0.1027 |
| **INL_TV ^a^** | 0.9608 ± 0.05389 | 0.9675 ± 0.05898 | 0.0607 ± 0.06285 | 0.9950 ± 0.05437 |
| **OPL_TV^a^** | 0.8377 ± 0.07128 | 0.7988 ± 0.04612 | 0.8071 ± 0.0759 | 0.8033 ± 0.04325 |
| **ONL_TV^a^** | 1.789 ± 0.1895 | 1.881 ± 0.1671 | 1.714 ± 0.1626 | 1.724 ± 0.2218 |
| **RPE_TV^a^** | 0.4126 ± 0.03541 | 0.4363 ± 0.02925 | 0.4164 ± 0.03177 | 0.4150 ± 0.04694 |
| **pRNFL-G^b^** | 98.78 ± 10.73 | 96.13 ± 5.743 | 100.2 ± 8.222 | 104.8 ± 9.865 |
| **pRNFL-PMB^b^** | 52.22 ± 8.600 | 55.50 ± 10.50 | 55.42 ± 9.060 | 51.00 ± 10.85 |
